# Anti-CfaE nanobodies provide broad cross-protection against major pathogenic enterotoxigenic *Escherichia coli* strains, with implications for vaccine design

**DOI:** 10.1038/s41598-021-81895-0

**Published:** 2021-02-02

**Authors:** Alla Amcheslavsky, Aaron L. Wallace, Monir Ejemel, Qi Li, Conor T. McMahon, Matteo Stoppato, Serena Giuntini, Zachary A. Schiller, Jessica R. Pondish, Jacqueline R. Toomey, Ryan M. Schneider, Jordan Meisinger, Raimond Heukers, Andrew C. Kruse, Eileen M. Barry, Brian G. Pierce, Mark S. Klempner, Lisa A. Cavacini, Yang Wang

**Affiliations:** 1 MassBiologics, University of Massachusetts Medical School, 460 Walk Hill Road, Boston, MA USA; 2 Department of Biological Chemistry and Molecular Pharmacology, Blavatnik Institute, Harvard Medical School, Boston, MA USA; 3 QVQ Holdings BV, Utrecht, The Netherlands; 4 Center for Vaccine Development, University of Maryland School of Medicine, Baltimore, MD USA; 5 University of Maryland Institute for Bioscience and Biotechnology Research, Rockville, MD USA

**Keywords:** Infectious diseases, Bacterial infection, Biotechnology, Drug discovery, Vaccines

## Abstract

Enterotoxigenic *Escherichia coli* (ETEC) is estimated to cause approximately 380,000 deaths annually during sporadic or epidemic outbreaks worldwide. Development of vaccines against ETEC is very challenging due to the vast heterogeneity of the ETEC strains. An effective vaccines would have to be multicomponent to provide coverage of over ten ETEC strains with genetic variabilities. There is currently no vaccine licensed to prevent ETEC. Nanobodies are successful new biologics in treating mucosal infectious disease as they recognize conserved epitopes on hypervariable pathogens. Cocktails consisting of multiple nanobodies could provide even broader epitope coverage at a lower cost compared to monoclonal antibodies. Identification of conserved epitopes by nanobodies can also assist reverse engineering of an effective vaccine against ETEC. By screening nanobodies from immunized llamas and a naïve yeast display library against adhesins of colonization factors, we identified single nanobodies that show cross-protective potency against eleven major pathogenic ETEC strains in vitro. Oral administration of nanobodies led to a significant reduction of bacterial colonization in animals. Moreover, nanobody-IgA fusion showed extended inhibitory activity in mouse colonization compared to commercial hyperimmune bovine colostrum product used for prevention of ETEC-induced diarrhea. Structural analysis revealed that nanobodies recognized a highly-conserved epitope within the putative receptor binding region of ETEC adhesins. Our findings support further rational design of a pan-ETEC vaccine to elicit robust immune responses targeting this conserved epitope.

## Introduction

Enterotoxigenic *Escherichia coli* (ETEC) is one of the most common causes of diarrheal illness in children under five years of age, adults in the developing world and in travelers to endemic areas. Based on WHO reports, ETEC-related diarrhea is one of the leading cause of death in the children under five years old in developing countries. Even if a child survives ETEC infection, there are long lasting effects throughout life including malnutrition, impaired cognitive development, learning disabilities and increased risk of non-communicable diseases in the adulthood^1–5^. ETEC infections are characterized by diarrhea, vomiting, stomach cramps, and in some cases mild fever. An estimated 10 million cases per year occur among travelers and military personnel deployed in endemic regions^3^. When adult travelers develop ETEC diarrhea, a short course of antibiotics can reduce the duration and volume of diarrhea. However, ETEC strains are becoming increasingly resistant to antibiotics^4^.

ETEC is a non-invasive pathogen that mediates small intestine adherence through bacterial surface structures that are known as colonization factors (CFs). Once bound to the small intestine, the bacteria produce toxins causing a net flow of water from enterocytes, leading to watery diarrhea*.* Development of an effective vaccine against ETEC bacterial attachment and colonization has long been considered as a promising approach against ETEC diarrhea. CFA/I is one of the most prevalent CFs and is composed of CfaE, the tip minor adhesin subunit, and a homopolymeric structural subunit, CfaB. In human clinical trials, oral administration of anti-CfaE bovine IgG provided protection against virulent ETEC challenge in over 60% of the test group, suggesting that an adhesin-based vaccine could be effective to elicit endogenous production of protective antibodies^6^. Recent studies in our laboratories demonstrated that when administered orally to mice and non-human primates, anti-CfaE human monoclonal antibodies inhibited bacterial colonization in the small intestine and protected animals from diarrhea associated with ETEC infection^7,8^.

ETEC strains are antigenically diverse with over 25 types of CF and coli surface (CS) antigens having been identified^4,9–11^. CFA/I represent the archetype of class 5 fimbriae, the largest class of human specific CFs that causes a majority of moderate to severe ETEC diarrheal cases^10–12^. Class 5 fimbriae includes three sub-classes, 5a (CFA/I, CS4, CS14), 5b (CS1, CS17, CS19, PCF071), and 5c (CS2). Other non-class 5 fimbriae have also been implicated in endemic and traveler diarrheal diseases including helical CS5, fibrillary CS3, CS21 and non-fimbrial CS6 adhesins^13^. As such, the development of a broadly protective and likely multicomponent vaccine against all major pathogenic ETEC strains remains one of the most important challenges in vaccine design^14,15^. In theory, a cross-protective vaccine could be generated with a combination of intact CFs^16^. However, such strategies could lead to unexpected instability or safety concerns, and high costs for manufacturing production. Despite advances in anti-ETEC vaccine research, there remains no licensed vaccine against ETEC.

The discovery of camelid heavy-chain variable domains (VHHs or nanobodies) has produced a new application for antibody-derived biologics^17^. Nanobodies consist of a highly stable and soluble single antigen-binding variable region (15 kDa), since they are derived from heavy chain only antibodies with no associated light chains^18^. Unlike conventional antibodies, nanobodies can access distinct antigenic sites (e.g., enzyme active sites, recessed regions of viral glycoproteins) due to smaller paratope diameters and a longer complementarity-determining region 3 (CDR3) that is able to form finger-like extensions^17,19^. Based on their unique characteristics, nanobodies can recognize conserved epitopes on hypervariable pathogens, such as HIV, poliovirus, norovirus, and coronavirus^20–24^. Consequently, nanobodies are being studied in clinical trials for the treatment of a wide range of diseases by both systemic and oral administration due to their excellent solubility and thermostability^25–27^.

In this study, we identified a panel of nanobodies that showed broad protective activities against eleven major types of disease-causing strains of ETEC. Oral administration of the selected nanobodies protected mice from colonization by 5 different strains of ETEC tested in vivo. Structural analysis revealed highly conserved epitopes shared by the nanobodies within the putative receptor binding region of ETEC adhesins. Due to the broad strain coverage of nanobodies discovered in this study, they could be used as prophylactic/therapeutic agents against diarrhea caused by multiple ETEC strains. In addition, the conserved epitope described in this study could be used for structure-based design of a pan-ETEC vaccine.

## Results

### Identification of anti-ETEC nanobodies from immunized llama and yeast display library

**Figure 1:**
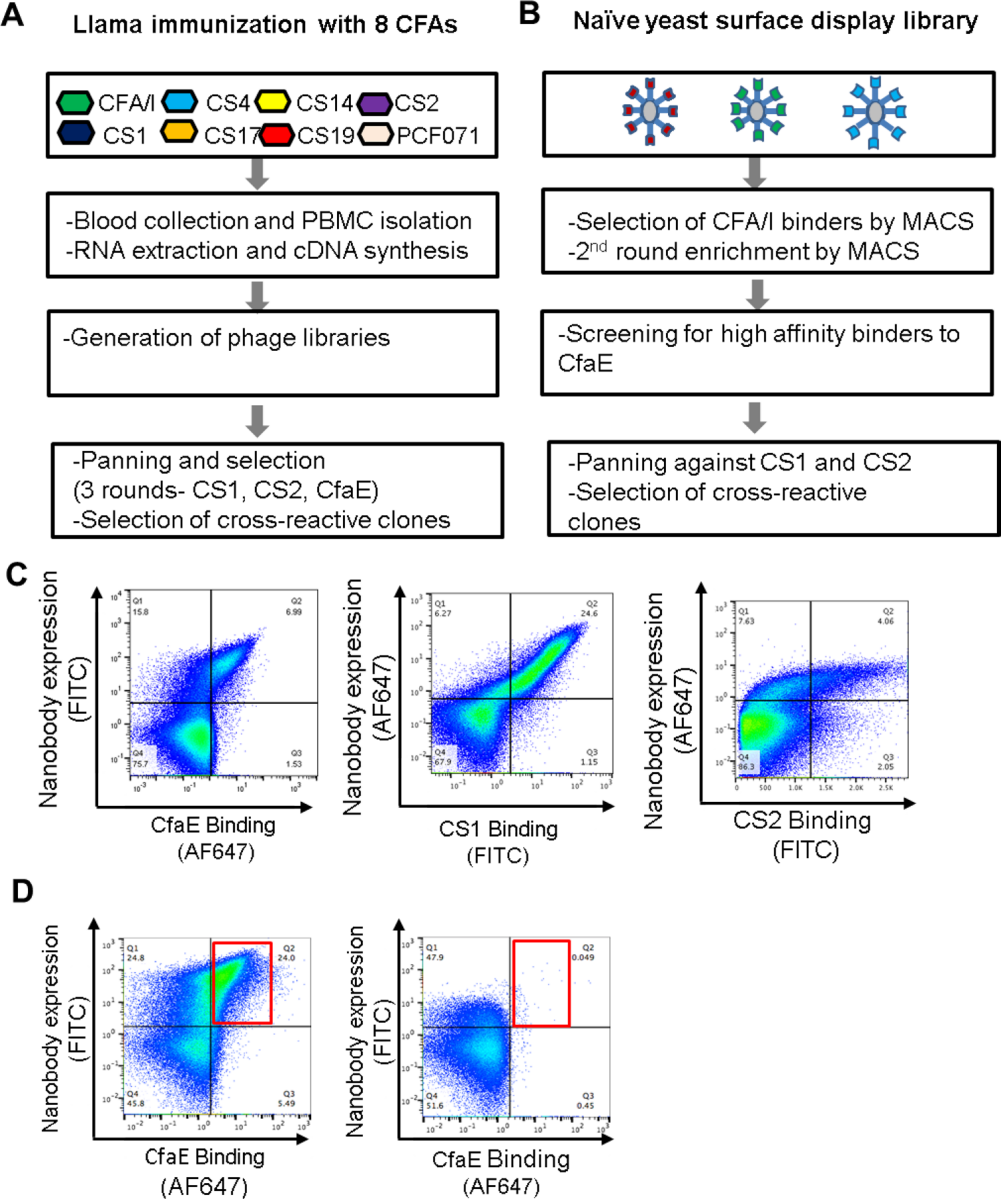
Identification of anti-ETEC nanobodies from immunized llama and yeast display library. (**A**) Flowchart of llama immunization and panning. Two male llamas were subcutaneously immunized with N-terminal fragments of eight class 5 ETEC adhesins followed by PBMC isolation and generation of phage-displayed libraries of 10^8^ in size. Libraries were panned on immobilized colonization factor antigens CS1 and CS2. Output from those pannings was used for a subsequent panning round on CfaE. Cross-reactive clones were sent for sequence determination and further characterization. (**B**) Flowchart of selection process of synthetic nanobodies. Binders to CfaE from the naïve yeast-displayed library were enriched over two rounds of selection by performing magnetic-activated cell sorting (MACS) on yeast that were stained with purified and labeled CfaE. High affinity binders were selected during fluorescence-activated cell sorting (FACS) and single cell clones were screened for binding with CS1 and CS2. Cross-reactive clones were sent for sequence determination and further characterization. (**C**) Flow analysis demonstrating example of binding of yeast displaying nanobody 2R23 to CfaE, CS1 and CS2 proteins. (**D**) Flow analysis demonstrating functional human monoclonal antibody 68-61 competing for binding to CfaE with yeast library derived nanobody 2R215 on yeast.

To generate nanobodies with broad cross-reactivity against ETEC adhesins, two male llamas were subcutaneously immunized with N-terminal fragments of eight class 5 ETEC adhesins (Fig. 1A). Serum response to adhesins was measured by ELISA and PBMC were isolated to generate phage-displayed libraries of size around 10^8^ as previously described^28^. Libraries were initially used for panning on immobilized colonization factor antigens CS1 and CS2, because of their relatively low sequence homology. Output from those pannings were used for a subsequent panning round on CfaE. Periplasmic extracts were prepared and tested for binding to all eight adhesin antigens in ELISA. Cross-reactive clones were sent for sequence determination and further characterization. From these selections, 26 candidate nanobodies were selected.

In parallel, we used a recently developed library of synthetic nanobodies displayed on the surface of *Saccharomyces cerevisiae*^29^. Two rounds of magnetic activated cell sorting (MACS) were performed using FITC and AlexaFluor647-labeled N-terminal fragments of CfaE to identify CfaE binding clones (Fig. 1B). High-affinity binders were enriched by fluorescent activated cell sorting (FACS) with decreasing concentration of FITC labeled CfaE as previously described^29,30^. Approximately 300 yeast clones were recovered from the yeast library screen. Binding of selected clones to antigen was verified by yeast surface staining against AlexaFluor647 labeled CfaE using flow cytometry. The clones showed a range of binding activities to CfaE (Supplemental Figure 1). All binders were subjected to additional rounds of screening for cross-reactivity against two other class 5 adhesins, CS1 and CS2 (Fig. 1C). A total of 30 nanobodies with cross-reactivity and unique sequences were selected for further characterization. In addition, a competitive FACS screen was performed against a potent anti-CfaE full length antibody, 68-61, to identify nanobodies specific to the receptor binding region of CfaE^8^. One clone, 2R215, was found to recognize an overlapping epitope with 68-61 (Fig. 1D, Supplemental Figure 2).

### Selected anti-CfaE nanobodies show cross-reactivity against all class 5 colonization factors

**Figure 2:**
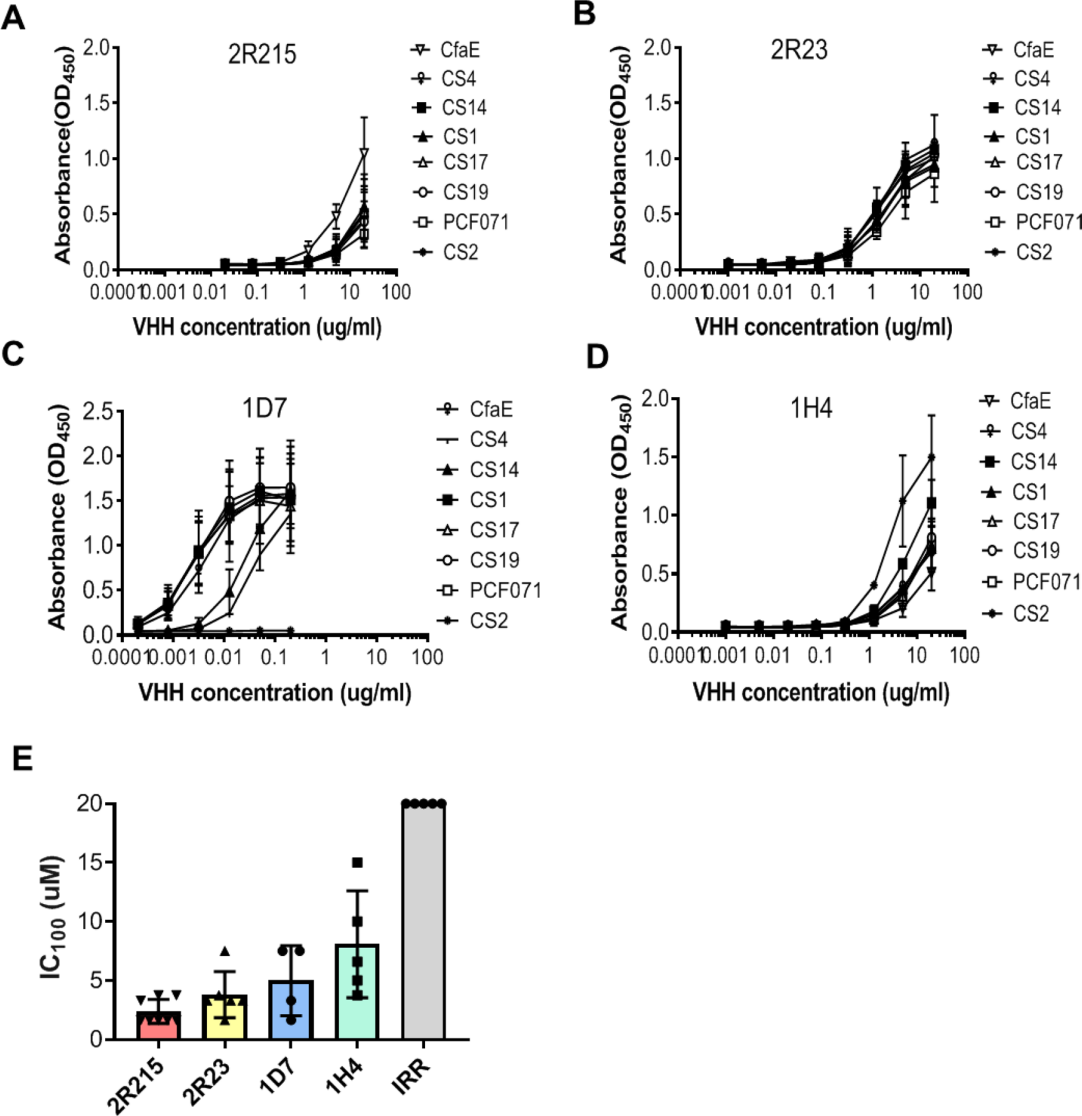
Selected anti-CfaE nanobodies show cross-reactivity against class 5 colonization factors. (**A**–**D**) Two yeast-derived clones (2R215 and 2R23) and two llama-derived clones (1D7 and 1H4) were examined by ELISA for binding to eight class 5 adhesins. These clones showed broad reactivity against multiple adhesins. Three biological replicates were performed. (E) The four lead nanobodies were tested for inhibition of mannose resistant hemagglutination (MRHA) against an ETEC strain expressing CFA/I (H10407). All nanobodies showed maximal inhibitory concentration (IC_100_) in the micromolar concentration range (2.4–8 µM). Four to eight biological replicates were performed.

To test the breadth of cross-reactivity, selected llama- and yeast-derived nanobodies were sequenced, cloned in pET-26b vector, expressed in *E.coli*, and examined by ELISA for binding to eight class 5 adhesins. Two yeast-derived clones (2R215 and 2R23) and two llama-derived clones (1D7 and 1H4) were selected based on their broad reactivity against multiple adhesins (Fig. 2A–D). The four lead nanobodies were then tested for inhibition of mannose resistant hemagglutination (MRHA) against an ETEC strain expressing CFA/I (H10407), the most prevalent class 5 adhesin. All nanobodies showed maximal inhibitory concentration (IC_100_) in the micromolar concentration range (2.4–8 µM) (Fig. 2E). We next examined if these nanobodies had activity against ETEC strains expressing six other class 5 adhesins in MRHA assay. Consistent with ELISA binding data, the four nanobodies were cross-protective against all six strains with an IC_100_ activity ranging from 0.4125 to 13.3 µM (Supplemental Table 1). We next examined the MRHA activity of nanobodies against ETEC strains expressing other pathogenic fimbrial and non-fimbrial adhesins including helical CS5, fibrillary CS3, and non-fimbrial CS6 adhesins, as well as another common diarrhea causing adhesin CS21. Surprisingly, four nanobodies showed broad protection against all tested strains with an IC_100_ ranging from 0.52 to 10 µM (Supplemental Table 1).

### Anti-CfaE VHH prevent ETEC colonization in the small intestine of a mouse model

**Figure 3:**
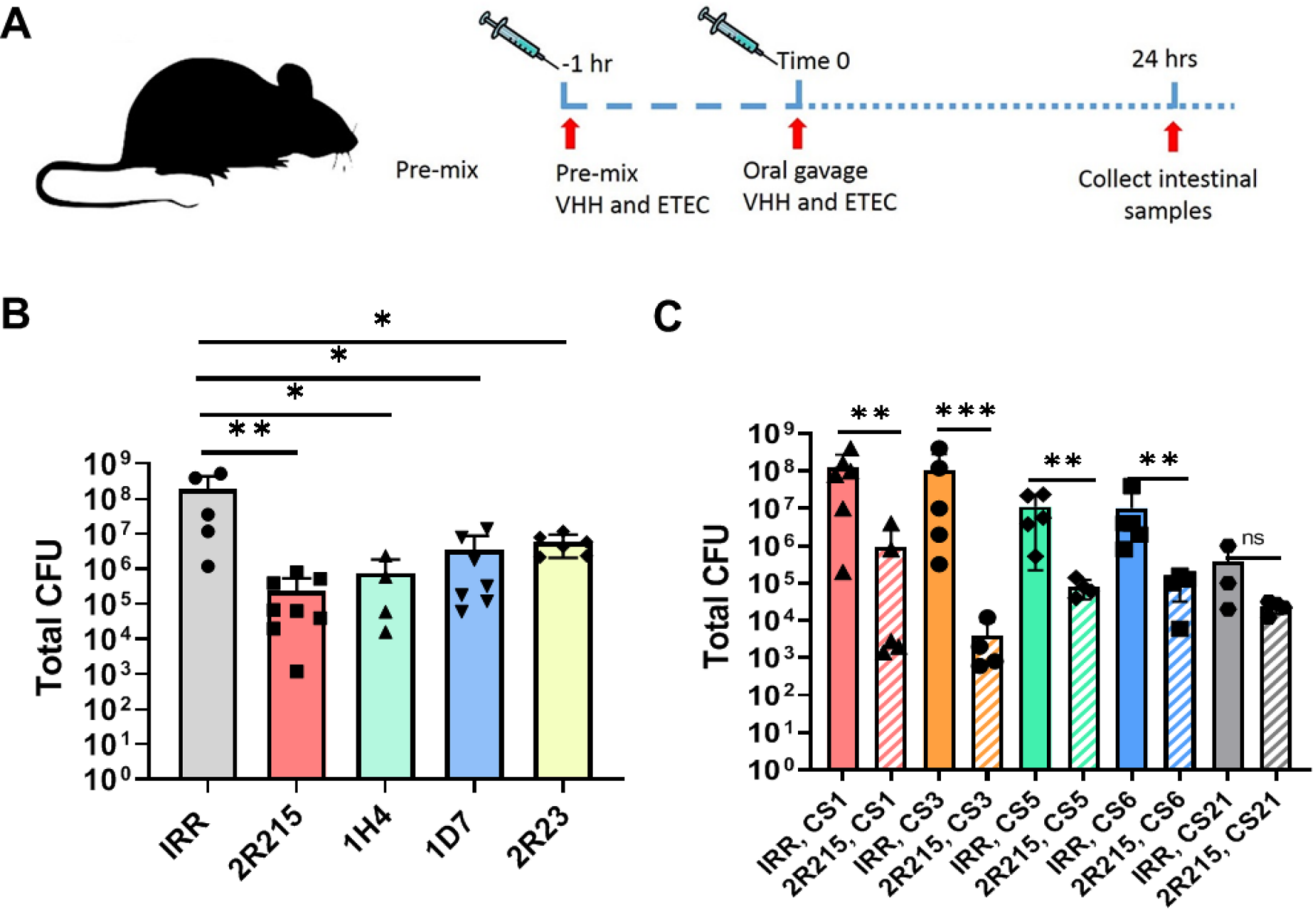
Anti-CfaE VHH prevent ETEC colonization in the small intestine of a mouse model. (**A**) Cartoon representation of mouse colonization experiment. VHH are pre-incubated with 10^7^ colony forming units (CFU) of ETEC 1 h prior to intragastric challenge. Animals are euthanized 24 h after challenge, and bacterial colonies in the small intestine are counted. (**B**) DBA/2 mice were challenged intragastrically with 10^7^ CFU of H10407 pre-incubated with 100 mg/kg of VHH. For 2R23 50 mg/kg of VHH was used due to lower production yields. At least 5 animals were tested for each condition. Results for all VHH were significantly different from those for the irrelevant VHH (*P* < 0.01 for 2R215 and *P* < 0.05 for 2R23, 1D7 and 1H4). Two biological replicates were performed. (**C**) DBA/2 mice were challenged intragastrically with 10^7^ CFU of strains expressing adhesins CS1, CS3, CS5, CS6, and CS21 pre-incubated with 100 mg/ml of 2R215. Treatment with 2R215 resulted in significant reduction of CFUs against ETEC strains expressing CS1 (2.1 log, *P* < 0.01), CS3 (4.4 log, *P* < 0.001), CS5 (2.2 log, *P* < 0.01) and CS6 (2 log, *P* < 0.01), but not the strain expressing CS21 (1.2 log, *P* > 0.05). Two biological replicates were performed for strain expressing CS1 adhesin. One biological replicate was performed for all other strains.

The lead nanobodies were further evaluated in a mouse model of ETEC colonization as described previously^8^ (Fig. 3A). Groups of five DBA/2 mice were given a mixture of bacteria and anti-CfaE nanobodies by oral gavage. All nanobodies were tested at 100 mg/kg, except for 2R23, which was tested at 50 mg/kg due to the low production yield. Twenty-four hours after challenge, the mice were euthanized and colony forming units (CFUs) in the small intestine were counted to determine the level of bacterial colonization. The efficacy of the anti-CfaE nanobodies was assessed by determining whether nanobodies could prevent adhesion/colonization of bacteria to the small intestine compared to an irrelevant nanobody control. In the anti-CfaE nanobody treatment groups, there was a log reduction of CFUs by 2.9 for 2R215, 2.3 for 1H4, 1.7 for 1D7 and 1.5 for 2R23 compared to the irrelevant nanobody control group (*P* < 0.01 for 2R215 and *P* < 0.05 for 2R23, 1D7 and 1H4). These results indicate that all four nanobodies showed significant activity in preventing colonization by H10407 strain at a dose of 50 or 100 mg/kg (Fig. 3B).

The lead nanobody, 2R215, was then tested against pathogenic ETEC strains expressing other adhesins that are commonly isolated from patients with ETEC-associated diarrheal diseases, including CS1, CS3, CS5, CS6, and CS21^11,13^. 2R215 demonstrated broad protection against all tested strains. Compared to the irrelevant control, treatment with 2R215 at 100 mg/kg resulted in significant reduction of CFUs against ETEC strains expressing CS1 (2.1 log, *P* < 0.01), CS3 (4.4 log, *P* < 0.001), CS5 (2.2 log, *P* < 0.01) and CS6 (2 log, *P* < 0.01), but not the strain expressing CS21 (1.2 log, *P* > 0.05) (Fig. 3C).

### Multimerization enhances the potency of 2R215 in vitro and in vivo

**Figure 4:**
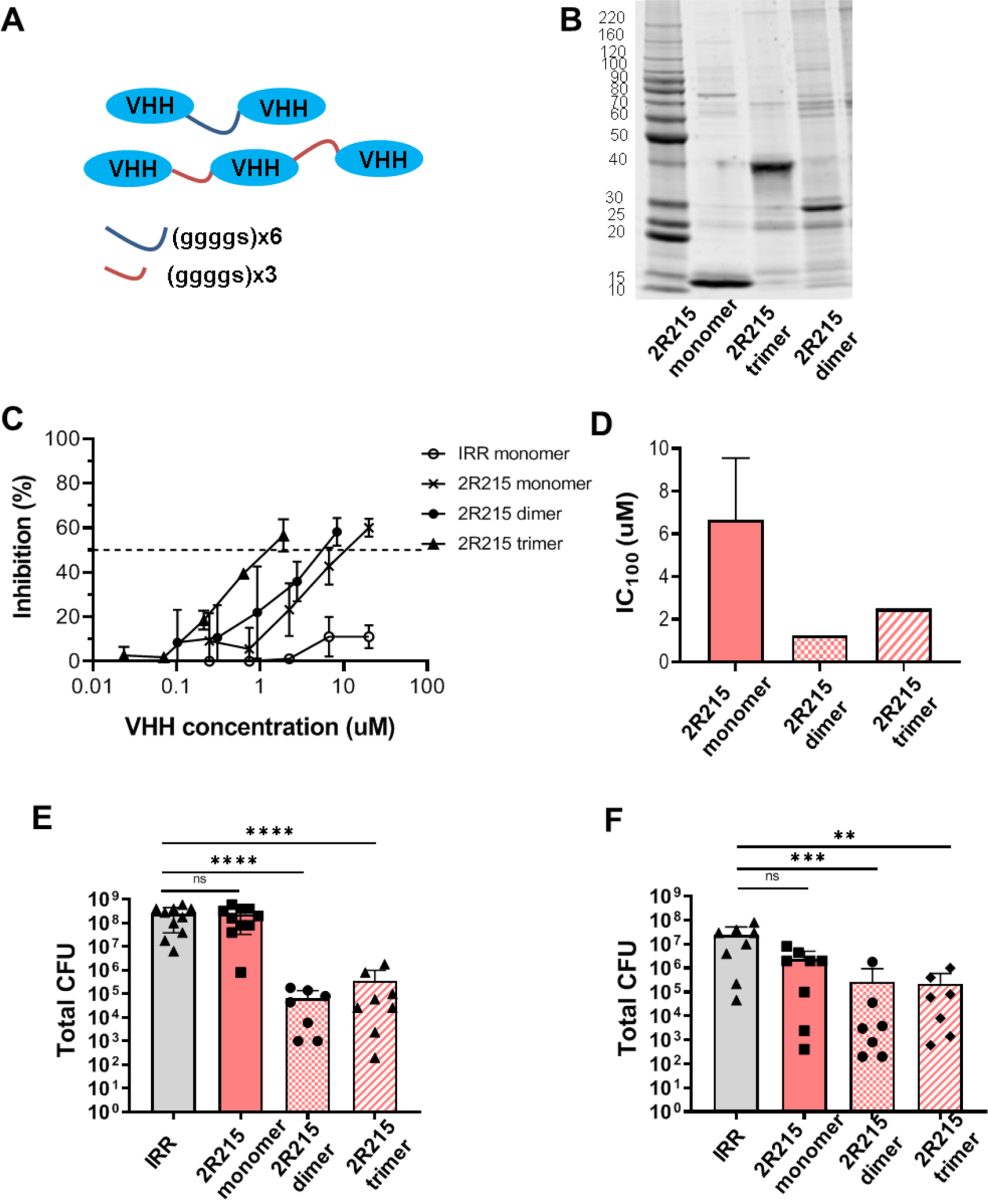
Multimerization enhances the potency of 2R215 in vitro and in vivo. (**A**) Cartoon representation of multimerization strategy. VHH 2R215 was engineered as a dimer or trimer molecules using 3 × or 6 × (G4S) linkers in tandem N to C terminus orientation. (**B**) Multimerized nanobodies were expressed in bacteria and purified using fused His tags. Purified multimer sizes were verified by polyacrylamide gel electrophoresis. (**C**) Dimeric and trimeric 2R215 show increased potency in Caco2 cell adhesion assays. The IC_50_ values are 10.213 µM for monomeric 2R215, 5.09 µM for dimeric 2R215 and 1.06 µM for trimeric form of 2R215. Three biological replicates were performed (*P* < 0.05 and *P* < 0.01 for dimeric and trimeric forms compared to monomeric forms respectively. *P* > 0.05 when dimeric was compared to trimeric format). (**D**) Activity in the MRHA assay is improved with an IC_100_ of 6.7 µM for monomeric, 1.25 for dimeric and 2.5 for the trimeric form of 2R215. Three biological replicates were performed. (*P* < 0.01 and *P* < 0.05 for dimeric and trimeric forms compared to monomeric forms respectively. *P* > 0.05 when dimeric was compared to trimeric format). (**E**) Mice inoculated with ETEC pre-incubated with 10 mg/kg of dimeric or trimeric forms of 2R215 showed a log reduction of 3.5 and 2.8 in colonization (*P* < 0.0001) compared to the monomeric 2R215 treated group at the same dose. Two biological replicates were performed. (**F**) The dimeric and trimeric forms of 2R215 retained their activity when administered 2 h prior to bacteria challenge as compared to the monomeric form. Pre-treatment with dimeric and trimeric 2R25 reduced total CFUs by 1.9 log (*P* < 0.001) and 2 log (*P* < 0.01) respectively. Two biological replicates were performed.

Multimerization of single domain nanobodies can increase their stability and potency^28,31^. To improve the potency of identified cross protective nanobodies, 2R215 was engineered as a dimer or trimer molecules using 3 × or 6 × (G4S) linkers in tandem N to C terminus orientation (Fig. 4A). Multimerized nanobodies were expressed in bacteria and purified using fused His tags (Fig. 4B). Both dimeric and trimeric 2R215 showed increased potency in Caco2 cell adhesion assays relative to the respective monomer. The IC_50_ values were 10.213 µM for monomeric 2R215, 5.09 µM for dimeric 2R215 and 1.06 µM for trimeric form of 2R215. (Fig. 4C). Activity in the MRHA assay was also improved with an IC_100_ of 6.7 µM for monomeric, 1.25 for dimeric and 2.5 for the trimeric form of 2R215 (*P* < 0.01 and *P* < 0.05 for dimeric and trimeric forms respectively) (Fig. 4D).

The dimeric and trimeric forms of 2R215 were further characterized in a mouse colonization model. Mice inoculated with ETEC mixed with 10 mg/kg of dimeric or trimeric forms 2R215 showed a log reduction of 3.5 and 2.8 in colonization (*P* < 0.0001) compared to the monomeric 2R215 treated group at the same dose (Fig. 4E). Moreover, the dimeric and trimeric forms of 2R215 retained their activity when administered 2 h prior to bacteria challenge as compared to the monomeric form. Pre-treatment with dimeric and trimeric 2R25 reduced total CFUs by 1.9 log (*P* < 0.001) and 2 log (*P* < 0.01) respectively (Fig. 4F). These results indicated that multimerization of monomeric nanobodies result in improved intragastric stability and protective potency. It should be noted that our current multimerization strategy (using G4S linkers) was selected for proof of concept based upon previous studies^28^. We anticipate that multimerization with other linkers may provide greater stability or better potency, which we will investigate in future studies.

### IgA Fc fusion enhances in vivo potency of 2R215 and 1D7

**Figure 5:**
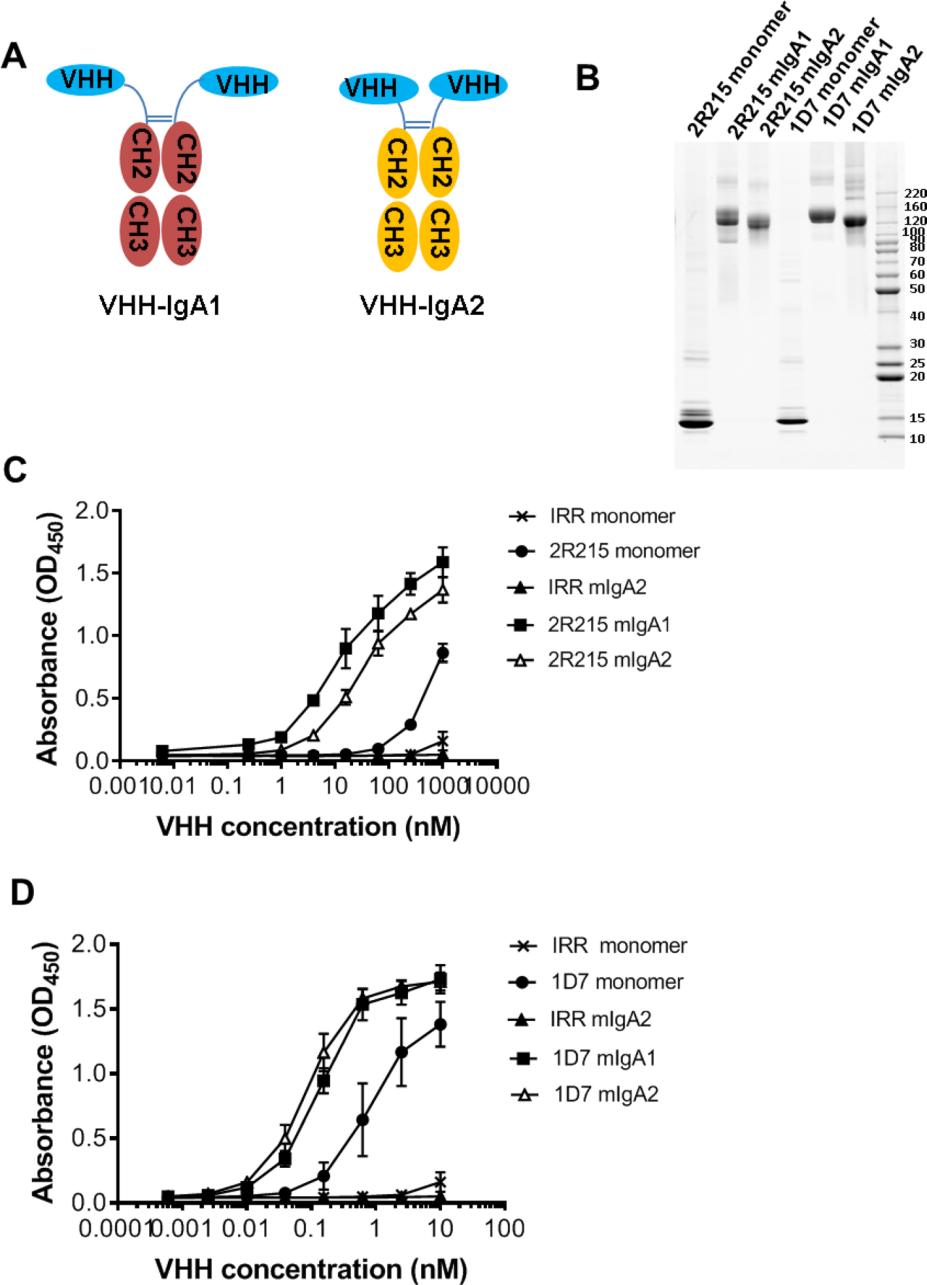
IgA Fc fusion enhances in vitro potency of 2R215 and 1D7. (**A**) Cartoon representation of IgA fusion strategy. To engineer VHH-IgA Fc fusionbodies, 2R215 and 1D7 were grafted on to the Fc domain of IgA1 and IgA2 (VHH-IgA1 and VHH-IgA2) at the hinge regions. (**B**) 2R215 and 1D7 VHH-IgA1 and VHH-IgA2 fusionbodies were expressed in Expi293 cells and the purified fusion protein sizes were verified by polyacrylamide gel electrophoresis. (**C**,**D**) ELISA showing the binding of fusionbodies 2R215 and 1D7 with CfaE. The binding activities of fusionbodies were enhanced as compared to monomeric nanobodies. Three biological replicates were performed.

**Figure 6:**
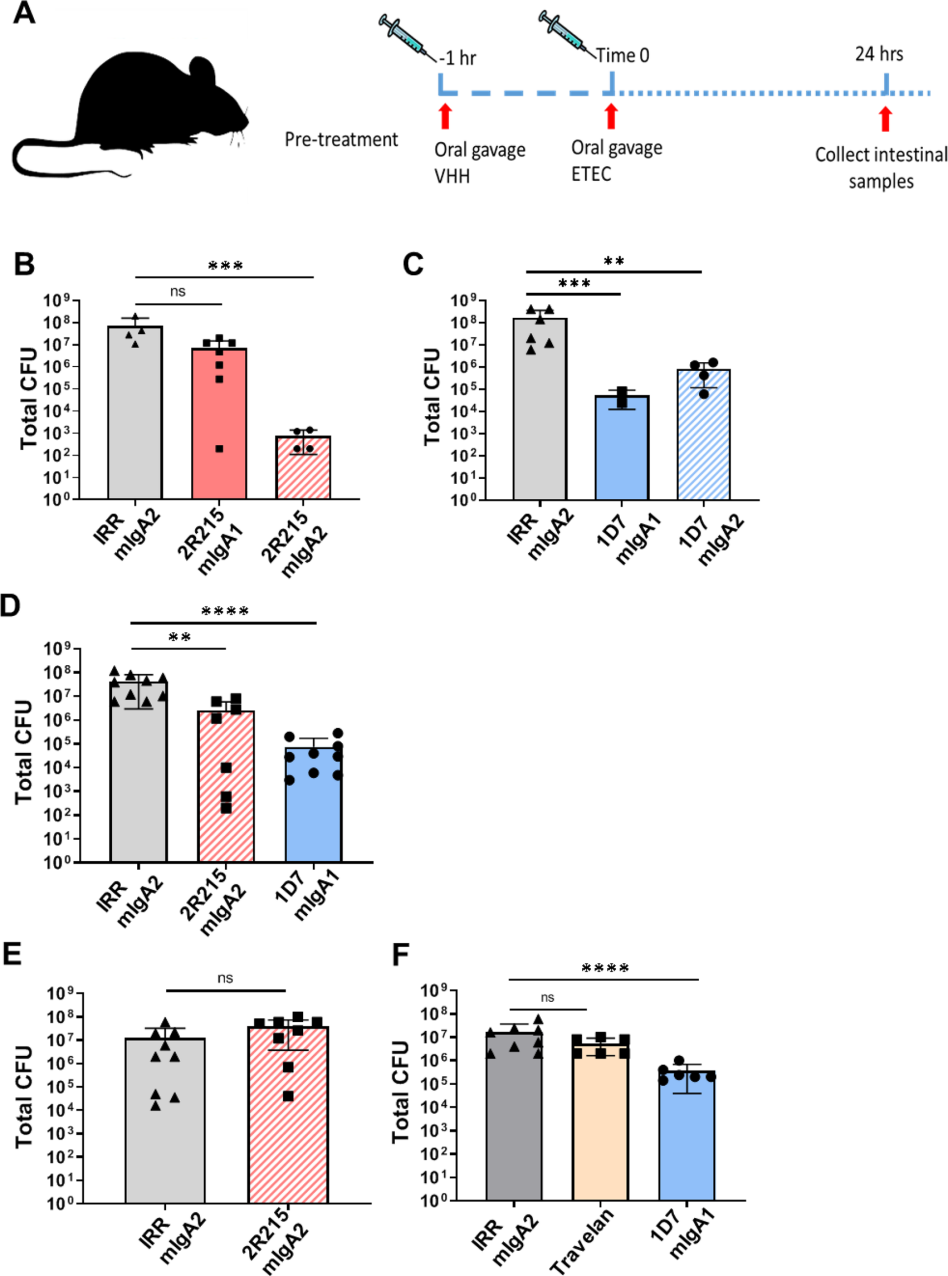
IgA Fc fusion enhances in vivo potency of 2R215 and 1D7. (**A**) Cartoon representation of pre-treatment protocol of VHH administration. VHH are administered one or two hours prior to administration of 10^7^ CFU of H10407. Animals are euthanized 24 h after challenge, and bacterial colonies in the small intestine are counted. (**B**,**C**) DBA/2 mice were challenged intragastrically with 10^7^ CFU of H10407 pre-incubated with 10 mg/kg of fusionbody. Treatment with 1D7 VHH-IgA1 resulted in 3.5 log reduction in CFU, *P* < 0.001) and 2R215 VHH-IgA2 in 5 log reduction in CFU (*P* < 0.001). (**D**) Fusionbodies 2R215 and 1D7 were intragastrically administered one hour prior to administration of 10^7^ CFU of H10407. Fusionbody pre-treatment resulted in reduction of CFUs by 1.2 log for 2R215 VHH-IgA2 (*P* < 0.01) and 2.7 log for 1D7 VHH-IgA1 (*P* < 0.0001). Two biological replicates were performed. (**E**,**F**) Fusionbodies 2R215 and 1D7 and Travelan were intragastrically administered two hours prior to administration of 10^7^ CFU of H10407. A 1.7 log reduction in total CFUs was observed in 1D7 VHH-IgA1 treated animals as compared to the group treated with irrelevant control (*P* < 0.0001). Two biological replicates were performed.

To gain effector function and improve mucosal stability, nanobodies could be engineered with the IgA Fc domain as IgA Fc fusionbodies (VHH-IgA) for oral administration^32^. To engineer VHH-IgA Fc fusionbodies, 2R215 and 1D7 were grafted on to the Fc domain of IgA1 and IgA2 (VHH-IgA1 and VHH-IgA2) at the hinge regions as bivalent VHH-Fc fusionbodies^33^ (Fig. 5A). 2R215 and 1D7 VHH-IgA1 and VHH-IgA2 fusionbodies were expressed in Expi293 cells and the purified fusion protein sizes were verified by polyacrylamide gel electrophoresis (Fig. 5B). The binding of fusionbodies with adhesin was maintained with increased binding activities as compared to monomeric nanobody 2R215 and 1D7 in ELISA (Fig. 5C,D). 2R215 and 1D7 fusionbodies were then tested in mouse colonization assay administered with ETEC strain H10407. While monomeric 2R15 and 1D7 inhibited colonization at 100 mg/kg (Fig. 3B), the IgA fusionbodies showed stronger inhibitory activity at a much lower dose of 10 mg/kg (Fig. 6A–D). Next, the most potent fusionbodies, 1D7 VHH-IgA1 (3.5 log reduction in CFU, *P* < 0.001) and 2R215 VHH-IgA2 (5 log reduction in CFU, *P* < 0.001), were examined in a pre-treatment model. Animals were orally inoculated with fusionbodies 1 h prior to ETEC challenge. Fusionbody treatment resulted in reduction of CFUs by 1.2 log for 2R215 VHH-IgA2 (*P* < 0.01) and 2.7 log for 1D7 VHH-IgA1 (*P* < 0.0001) (Fig. 6C,D). Furthermore, the 1D7 VHH-IgA1 fusionbody retained activity when the pre-treatment time was extended to 2 h. A 1.7 log reduction in total CFUs was observed in 1D7 VHH-IgA1 treated animals as compared to the group treated with irrelevant control (*P* < 0.0001) (Fig. 6E). We also compared the activity of the 1D7 VHH-IgA1 fusionbody to Travelan, a commercial hyperimmune bovine colostrum (HBS) product used for the prevention of ETEC-induced diarrhea. While Travelan showed activity in 1 h pre-treatment when used at 40 mg/kg (Supplemental Figure 3), it did not inhibit colonization after 2 h of pre-treatment (Fig. 6E).

### Epitope mapping of lead anti-ETEC VHHs

To elucidate the molecular basis of the interaction between anti-ETEC nanobodies and adhesins, we performed computational analysis and experimental mutagenesis to map nanobody binding determinants. Residues of interest on CfaE were identified through analysis of side chain surface exposure, sequence conservation, and docking simulations (BioLuminate, Schrödinger Release 2018-3, Schrödinger, LLC, New York, NY, 2016), and were mutated to alanine for experimental testing of effects on nanobody binding using ELISA. Most of the residues selected for mutagenesis are located in the putative receptor binding pocket, while other conserved and surface-exposed sites outside of that region were also tested.

**Figure 7:**
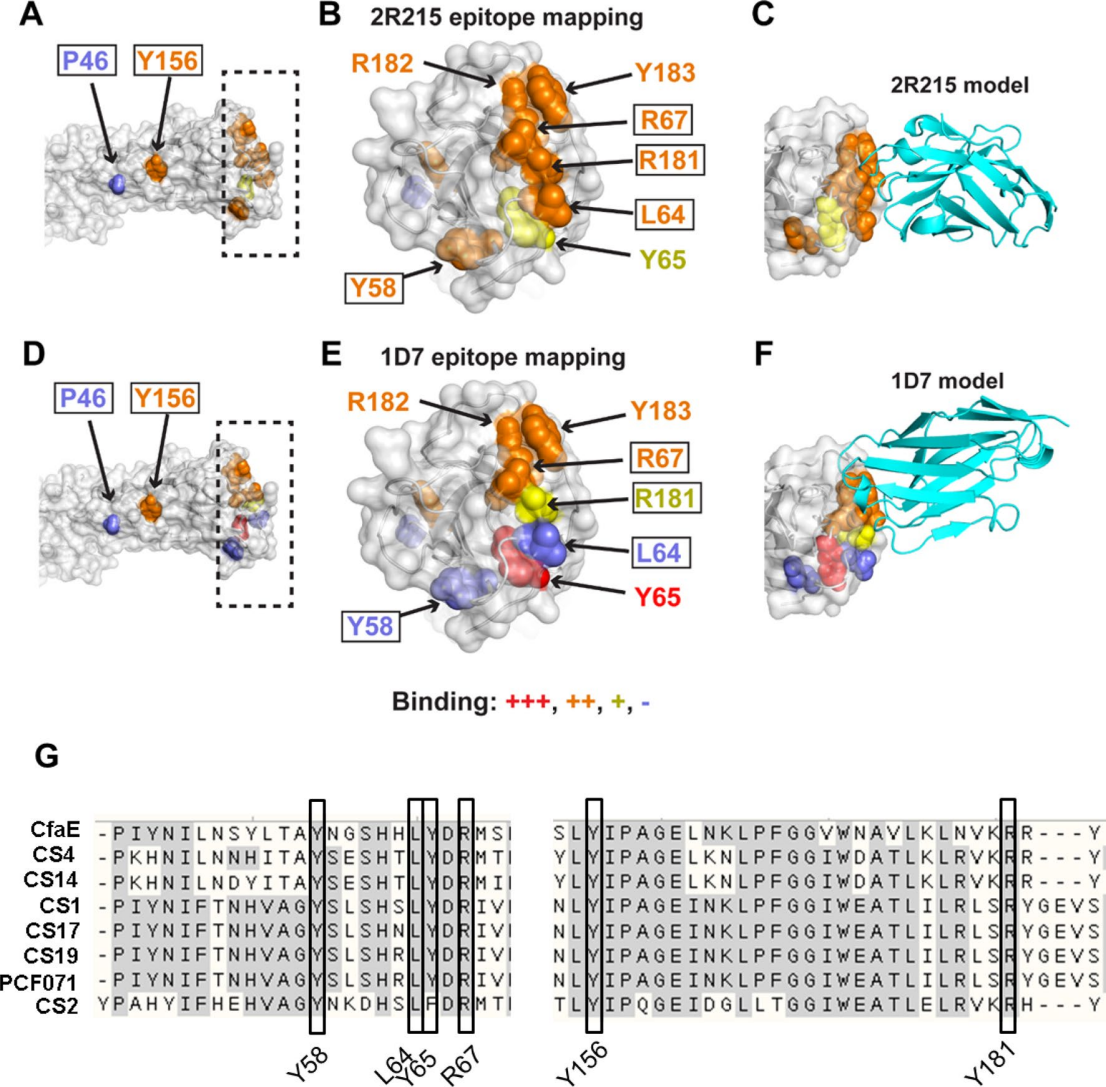
Epitope mapping of lead anti-ETEC VHHs. (**A**–**D**) Figures showing molecular structures were generated with PyMOL version 1.8 (Schrodinger, LLC, New York, NY, 2016, PyMOL | pymol.org). BioLuminate software [BioLuminate, Schrödinger Release 2016‐4, Schrödinger, LLC, New York, NY, BioLuminate^®^ | Schrödinger (schrodinger.com)] was used to perform initial modeling of nanobodies and docking simulations to CfaE. Binding effects of alanine substitutions are shown on the CfaE structure (PDB code 2HB0) for lead VHHs (**A**) 2R215 and (D) 1D7, with close-up of putative receptor binding region (boxed in **A**,**D**) shown in (**B**) and (**E**). CfaE is shown as gray surface and cartoon, with residues tested by alanine scanning shown as spheres and colored by measured binding effects of CfaE alanine substitutions: major (+++, red), moderate (++, orange), minor (+, yellow), none (−, blue). Values correspond to those in Supplemental Table 2. To provide comparison of shared or distinct binding determinants, only residues with alanine scanning binding data for all nanobodies are shown. Residues are labeled in (**A**), (**B**), (**D**), (**E**), and residues that are fully conserved across 8 fimbrial adhesins are boxed. Docking models of (C) 2R215 and (F) 1D7 VHHs bound to CfaE were generated using ABodyBuilder^44^ and ZDOCK^35^, with modeled bound VHHs shown as cyan cartoons. (**G**) Sequences of eight class 5 fimbrial adhesins were aligned using ClustalX algorithm. Inserts show regions in adhesion proteins where highly conserved residues (black box) required for nanobody binding are located.

Experimental binding affinity measurements revealed that several highly conserved residues in the putative receptor binding pocket are key determinants of nanobody binding (Supplemental Table 2). Binding effects of residue substitutions are shown on the CfaE structure for the lead nanobodies 2R215 and 1D7 in Fig. 7. Highly conserved receptor binding pocket residues R67 and R181 are shared determinants of 2R215 and 1D7 binding, in addition to residues R182 and Y183, the latter of which is well-exposed on the CfaE surface (Fig. 7B,E). Of note, R181 of CfaE was previously found to be critical for ETEC binding in a human in vitro organ culture^34^. The R181A substitution had a limited effect on nanobody 1D7 binding (< 20% disruption), suggesting less engagement of that residue relative to others in that region such as R67, R182 and Y183. Other residues, including L64 and Y65, showed varying 2R215 and 1D7 binding effects for alanine substitutions, indicating fine differences in nanobody-adhesin recognition. Alanine substitutions of conserved residues outside of the putative receptor binding region were also tested, with P46A leading to no effects on binding, while Y156A unexpectedly led to moderate loss of nanobody binding. Given its distance from the likely nanobody binding sites, the Y156A substitution likely has an allosteric effect on CfaE, leading to an alteration in the structure or dynamics in the putative receptor binding pocket. To view possible modes of CfaE engagement for the 2R215 and 1D7 nanobodies, we performed docking global simulations of the modeled nanobodies using ZDOCK^35^ and the unbound structure of CfaE (PDB code 2HB0). Based on clustering analysis, we identified models that agree with epitope mapping data, with 2R215 and 1D7 models targeting key binding residues on CfaE (Fig. 7C,F).

Sequence alignment revealed that most of these residues are highly conserved among at least eight class 5 adhesins^36^, which is consistent with our observation that these nanobodies are broadly cross protective against multiple ETEC strains (Fig. 7G).

## Discussion

ETEC strains are highly antigenically diverse, expressing over 25 types of adhesion factors. It has been very challenging to generate a pan-ETEC vaccine with broad coverage against diverse strains that cause diarrheal disease. In the present study, we used a llama immunization and a synthetic yeast library screen to identify and characterize a panel of nanobodies that recognize highly conserved epitopes on hypervariable adhesins. Among the 50 isolated nanobodies, four lead nanobodies showed broad coverage against 11 pathogenic ETEC strains and protected animals from infection.

Multimerization and IgA Fc fusion of nanobodies has emerged as an effective way to increase potency and mucosal half-life^28^. Recent studies have shown the feasibility of producing various formats of nanobodies in plants and microorganisms. High levels of VHH-IgA fusionbodies were effectively produced in yeast and soybean and have been proven efficient for mucosal protection against ETEC in piglets^33,37^. Moreover, an anti-rotavirus VHH (ARP1) produced in yeast and rice could protect mouse pups against rotavirus-induced diarrhea^38,39^. Oral administration of ARP1 produced in yeast was safe and effective in reducing diarrhea in male infants with severe rotavirus-associated diarrhea^40^.

In the absence of a pan-ETEC vaccine, our study provides the first proof of concept that oral administration of a nanobody, prior to or during infection, could confer broad protection against various ETEC strains. The panel of nanobodies described in this study showed broad cross-protection against 11 major disease causing ETEC strains in HAI assay and prevented colonization in vivo when challenged by five out of six different ETEC strains tested.

Additionally, in order to further expand breadth of ETEC strain coverage by nanobodies, a cocktail of two or more nanobodies could be used.

Compared to Travelan, a commercial HBC product used to prevent ETEC-induced diarrhea, 1D7 VHH-IgA1 fusionbody appears to be active in the mouse colonization model (1.7 log reduction in CFU) when administered two hours prior to ETEC challenge and is effective at a lower dose (10 mg/kg of fusionbody vs 40 mg/kg of Travelan).

Amenability of nanobodies to modifications and optimizations, and technological advances in large-scale manufacturing of biological proteins in plants and microorganisms will make nanobody-based immunotherapy a potent and cost-effective prophylaxis or treatment for ETEC.

We previously described a positively charged binding pocket at the N-terminal domain of CfaE important for ETEC interaction with the negatively charged sialic acid on the host epithelial cells^8^. This binding pocket is formed by three arginine residues, R67, R181, and R182, and a cluster of surrounding residues, which are all highly conserved among the class 5 fimbrial adhesins. Mutations of the arginine residues result in a complete loss of binding activity of ETEC to host cells^34^. The cross-protective nanobodies identified in this study were found to be focused on this critical site, providing a target for rational vaccine design to focus the immune response to this region. Recent work has highlighted the success of structure-based antigen design to elicit responses to key conserved epitopes, including for complex epitope structures^41^. Experimental structural determination of one or more of these nanobodies bound to a representative fimbrial adhesin can confirm mapping-based binding models and serve as the basis for rational ETEC vaccine design to generate immunogens to elicit robust responses targeting this conserved site.

## Methods

### Antigen cloning, expression, and purification

The nucleic acid sequences of N-terminal adhesin domains of CfaE and class 5 adhesins (GenBank M55661) were cloned into a pMAL-C5X vector (Addgene) in-frame with an MBP tag to express as periplasmic proteins with improved solubility (MBP–CfaE-N). The donor strand complement was included to ensure the overall protein expression and stability, as reported previously^42^. All cloned constructs were transformed into SHuffleT7 Competent *Escherichia* *coli* (NEB), and expression was induced with 1 mM IPTG (isopropyl-d-thiogalactopyranoside). Bacteria were lysed, and proteins were purified with amylose resin (NEB) and eluted with 20 and 50 mM maltose (Sigma).

### Llama immunization

Immunizations of llamas and library constructions were performed as described previously^28^. Briefly, two male llamas were immunized with antigen cocktails that included eight class 5 colonization factors (300 μg each antigen, 2400 μg total for each animal). The first cocktail consisted of CS2, CS4, CS14, CfaE and the second cocktail consisted of CS19, CS17, CS1, and PCF071. The two antigen cocktails were injected into each animal weekly for six weeks and 150 ml blood was collected for isolation of RNA from the peripheral blood lymphocytes after the last immunization on the 7th week. Good immune response was observed in both animals against all antigens. RNA isolation and library constructions were performed by amplifying nanobody genes and ligating into a phagemid vector. Two phage display libraries were generated with a size of approximately 5 × 10^8^ transformed *E. coli* TG1 bacteria and a correct insert ration of ~ 100%. Selection of target-binding nanobodies was performed by phage-display selections against immobilized antigen proteins on Maxisorp plates (NUNC, Thermo Fisher Scientific). The initial screening was performed against CS1 and CS2, followed by a second round of screening against the CfaE antigen. Phages were eluted by unspecific and competitive elutions. Eluted phage were used to infect exponentially growing *E. coli* TG1 that were plated on Luria broth (LB) agar plates containing 2% (w/v) glucose and 100 µg/ml ampicillin. In addition, eluted phages were serially diluted, infected in TG1 and 5 µl spots of these infections were plated to allow estimating how many phages were eluted. Periplasmic extracts were prepared according to standard protocols involving overnight production in 2YT medium and induction with IPTG. Extracts were tested for binding to all 8 Cfa antigens in ELISA. Based on HinfI digestion pattern, amino acid sequences and cross-reactive binding to Cfa antigens, unique candidate genes were selected for further characterization.

### Yeast library screening

Yeast library screening was performed as previously described^29^. For the first round of magnetic-activated cell sorting (MACS), 1 × 10^10^ of *S. cerevisiae* expressing a surface displayed library of synthetic nanobodies^29^ were centrifuged, resuspended in binding buffer (20 mM HEPES pH 7.5, 150 mM NaCl, 2.8 mM CaCl_2_, 0.05% MNG, 0.005% CHS, 0.1% BSA, 0.2% maltose) and then incubated with anti-fluorescein isothiocyanate (FITC) microbeads (Miltenyi) and FITC labeled MBP for 40 min at 4 °C. Yeast cells were passed through an LD column (Miltenyi) to remove any yeast expressing nanobodies which interacted with the microbeads or MBP. The remaining yeast cells that flowed through the column were centrifuged, resuspended in binding buffer, and incubated with 1 µM of FITC-labeled N-terminal CfaE protein for 1 h at 4 °C. Yeast cells were then centrifuged, resuspended in binding buffer with anti-FITC microbeads, and incubated for 15 min at 4 °C before passing them into a LS column (Miltenyi) and collecting the eluate enriched for CfaE-binding nanobodies. The eluted yeast cells were expanded and used in a subsequent round of MACS to further enrich for CfaE-binding nanobodies. The second round was performed similarly to the first but beginning with 4 × 10^8^ yeast and substituting FITC-labeled CfaE with AlexaFluor647-labeled CfaE, and anti-FITC microbeads with anti-AlexaFluor647 microbeads. High affinity binding yeast cells were isolated with FACS. In the first round of FACS, yeast binding to 300 nM of AlexaFluor488 labeled CfaE were collected. These yeast clones were grown and subjected to second round of FACS in the presence of human monoclonal antibody^8^ that was shown to bind in the proximity of the receptor binding domain. Clones that were outcompeted from binding to the antigen were collected. About 400 individual clones from two rounds of FACS were then grown, stained in a 96-well plate, assessed via flow cytometry for binding specificity to CfaE, and sequenced. 30 clones with unique sequences were isolated and chosen for further characterization, that included further panning with FITC-labeled class 5 adhesins CS1 (class5b) and CS2 (class5c).

### Nanobody purification

Nanobody sequences were cloned into pET-26b vector (EMD Millipore, Cat #69862) to express as a fusion protein with a C-terminal 6xHis tag. Sequence-verified clones were transformed into T7 Express lysY BL21 *E. coli*. Bacteria were grown in Terrific Broth containing 1 mM MgCl_2_ and 0.01% glucose to an OD_600_ = 0.7 before induction with 1 mM IPTG. Cells were harvested after overnight incubation at 27 °C. Following the osmotic shock, nanobodies were purified from the periplasmic fraction by Ni-NTA chromatography (Gold Biotechnology) and dialyzed against PBS to remove imidazole.

### ETEC strains

H10407 expressing CFA/I fimbriae was purchased from ATCC (ATCC 35401). ETEC strain H10407 was cultured on 2% agar containing 1% Casamino Acids (Sigma) and 0.15% yeast extract (Fisher Bioreagents) plus 0.005% MgSO_4_ (Sigma) and 0.0005% MnCl_2_ (Sigma) (CFA agar plates) overnight at 37 °C. A total of 1 × 10^8^ CFU/ml were resuspended in 20% glycerol (Sigma) in phosphate-buffered saline (PBS) solution and kept frozen at − 80 °C until needed. Strains expressing other adhesin molecules were obtain from University of Maryland. These strains included: #200145 (CS1), #201546 (CS2), #503046 (CS4), #400599 (CS14), #700056 (CS17), #204648 (CS19), #100483 (CS3), #204348 (CS5), #100001 (CS6) and #100171 (CS21).

The growth conditions are the same as H10407.

### Generation of multimerization and VHH-IgA fusion

Nanobodies were multimerized to dimeric or trimeric forms with (G4S) linkers. The dimeric forms were generated using 6 × (G4S) linker to connect two monomeric VHHs in tandem N terminus to C terminus orientation. Trimers were generated using two 3 × (G4S) linkers between monomeric nanobody units. The multimers were cloned into pET-26b vector adding a C-terminal 6xHis tag. Nanobodies were purified from the periplasmic fraction by Ni-NTA chromatography (Gold Biotechnology) and dialyzed against PBS to remove imidazole.

To generate VHH-IgA fusionbodies, monomeric nanobody sequences were cloned into a pcDNA 3.1 vector containing heavy chain constant region of IgA1 or IgA2 chains without the CH1 domain. Each vector was transformed in NEB5 competent cells, and sequences were verified ahead of transient transfection.

### ELISA

96-well plates (Nunc) were coated overnight at 4 °C with 2 µg/ml of purified MBP–CfaE-N. The plates were blocked with 1% BSA plus 0.05% Tween 20 in PBS for 1 h. Purified nanobodies were diluted in 1 PBS at a starting concentration of 20 μg/ml for 2R215, 2R23 and 1H4 and 0.2 μg/ml for 1D7, followed by 1:4 serial dilutions in PBS, and added to the plates for 1 h. The plates were stained with hydroxy peroxidase-conjugated rabbit anti-camelid IgG Fc (1:10,000) for 1 h and developed using TMB Peroxidase substrate (SeraCare). Absorbance at an optical density at 450 nm (OD_450_) was measured on an Emax precision plate reader (Molecular Devices).

### Flow cytometry

Binding of the yeast surface expressing nanobodies to fluorescently labeled antigens was determined as described previously^29^. Briefly, single clone pools were induced by galactose in Trp- media. 2 × 10^6^ cells were stained in selection buffer^29^ in the presence of anti-AlexaFluor647 labeled HA antibody to monitor clone expression, and FITC labeled antigens with the final concentration of 100 nM. To determine whether nanobody was competing with functional antibody, antibody 68-61 at a concentration of 100-fold of reported Kd was included in the staining mixture. Yeast cells were washed, resuspended in the selection buffer and subjected to flow cytometry on MACSquant.

### Mannose-resistant hemagglutination assay of human group A erythrocytes

H10407 strain cultures were taken from frozen cell banks and diluted in a sterile 0.15 M saline solution until an OD_600_ of 1 was reached for the assay. H10407 cell bank was used at 1:4 dilution.

Other ETEC strains except #400599 (CS14) were cultured in ETEC medium containing 1% Casamino Acids (Sigma) and 0.15% yeast extract (Fisher Bioreagents) plus 0.005% MgSO_4_ (Sigma) and 0.0005% MnCl_2_ (Sigma) and 0.5 mg/ml of Deferoxamine (Sigma Aldrich). CS14-expressing strain (#400599) was cultured on 2% agar containing 1% Casamino Acids (Sigma) and 0.15% yeast extract (Fisher Bioreagents) plus 0.005% MgSO_4_ (Sigma) and 0.0005% MnCl_2_ (Sigma) (CFA agar plates) overnight at 37 °C plates. Bacteria were resuspended in 1 × PBS and dilutions were used to determine the concentration needed to inhibit hemagglutination.

Type A-positive human erythrocytes stored in K3EDTA were washed three times with 0.15 M saline solution and resuspended in the same solution to a final concentration of 1.5% (vol/vol). In a U-bottom 96-well plate (Nunc Thermo Scientific), 100 µl of nanobody was added in duplicate to the top row and diluted 1:2 down the plate in a 0.15 M saline solution. Fifty microliters of appropriately diluted ETEC was added to each well together with 50 µl of a 0.1 M d-mannose solution (Sigma). The plate was incubated for 10 min at room temperature. After incubation, 50 µl of blood solution was added to the plate and mixed well (200 µl final volume). Plates were allowed to sit stagnant at 4 °C for 2 h. Hemagglutination was then observed without the aid of magnification. The absence of a pellet of erythrocytes at the bottom of the well is indicative of positive hemagglutination. Blood was ordered fresh every other week from BioIVT- non-blood bank provider of human and animal biological matrices.

### Caco-2 adhesion assay

Caco-2 cells seeded at 1 × 10^4^ cells/ml were grown in 96-well tissue culture plates containing Dulbecco’s modified Eagle’s medium (DMEM), at 37 °C in 5% CO_2_ statically. H10407 strain of ETEC was grown overnight at 37 °C in ETEC medium containing 1% Casamino Acids (Sigma) and 0.15% yeast extract (Fisher Bioreagents) plus 0.005% MgSO_4_ (Sigma) and 0.0005% MnCl_2_ (Sigma). The next day, bacteria were resuspended in PBS and diluted until an OD_600_ nm of 0.4 was reached. Antibody dilutions were set up in a deep well plate. Antibody dilutions and bacteria were combined at a 1:10 ratio and allowed to shake at 300 rpm for 1 h at room temperature. Meanwhile, Caco-2 cells were washed and incubated in antibiotic free DMEM containing 500 µg/ml of a nanobody. After incubation, 0.035 ml of the mixture of antibody and bacteria was added to each well containing Caco-2 cells. The cells were then incubated statically for 3 h at 37 °C. The cells were then washed four times with 1 ml PBS to remove non adherent ETEC cells and intensity of luciferase signal was determined.

### Mouse intestine colonization assays

All methods used in animal work were carried out in accordance with relevant guidelines and regulations.

All experimental protocols were approved by UMASS Medical School Institutional Animal Care and Use Committee (IACUC) and Institutional Bio-Safety Committee (IBC).

Six- to eight-week-old DBA/2 mice were pretreated with streptomycin (5 g/l) in the drinking water for 24–48 h. Twelve hours prior to bacterial administration, the water bottle was replaced with regular drinking water. One hour prior to bacterial administration, mice received cimetidine (50 mg/kg) intraperitoneally to reduce the effect of stomach acid on ETEC. A total of 1 × 10^7^ CFU of ETEC strains diluted in PBS were incubated with 100 mg/kg of monomeric anti-CfaE nanobody or an irrelevant nanobody 1 h prior to challenge. In pre-mix model, bacteria and nanobody were mixed and administered in 200 µl volume by oral gavage using 20-gauge bulb-tip feeding needles. In the pre-treatment model, mice were pre-treated with nanobody in 200 µl volume in PBS via oral gavage. One or two hours later, bacteria were administered in 100 µl PBS by oral gavage. The mice were allowed to survive for 24 h. At 12 h before euthanasia, food was withdrawn. Following isolation of the small intestine, two segments of the ileum (3 cm each), beginning within 0.5 cm of the ileocecal junction and extending proximally 6 cm, were removed and placed in 1 ml sterile PBS^43^. Tissues were mechanically homogenized. Samples were serially diluted on MacConkey agar plates and incubated overnight at 37 °C. Bacterial CFU were counted the next day.

### Epitope mapping

Alanine mutants of CfaE residues were cloned individually by Genscript into pMAL-C5x vector, and the resulting constructs were transformed, expressed, and purified as described above. ELISA was performed to determine the binding of the nanobodies to the mutant proteins in comparison to that of the wild type.

### Molecular modeling

BioLuminate software (BioLuminate, Schrödinger Release 2018-3, Schrödinger, LLC, New York, NY) was used to perform initial modeling of nanobodies and docking simulations to CfaE. Subsequent modeling of nanobody structures was performed using the ABodyBuilder antibody modeling server^44^ (with default parameters) followed by docking simulations using the ZDOCK server^35^ with default ZDOCK protocol (version 3.0.2), and the structure of unbound CfaE (PDB code 2HB0). To avoid docking models with nanobodies bound to the CfaE C-terminal domain, CfaE residues 200 and above were blocked on the server. ZDOCK server models were post-processed using the Fraction of Common Contacts (FCC) clustering algorithm^45^ to identify prevalent predicted nanobody-CfaE recognition modes among all 2000 ZDOCK models for each complex, using a contact identity cutoff of 0.75 as suggested by FCC documentation. Manual inspection of exemplars from most populous clusters for each interaction, to compare CfaE interface residues of models with epitope mapping data, was used to select docking model candidates. Figures showing molecular structures were generated with PyMOL version 1.8 (Schrodinger, LLC, New York, NY).

### Statistical analysis

Statistical calculations were performed using the software Prism version 7.03 (GraphPad Software, La Jolla, CA). Comparisons in colonization experiments were performed using one-way analysis of variance (ANOVA) with Bonferroni correction for post hoc multiple comparisons.

## Supplementary Information

Supplementary Information.
