# Supplementary material for: Anti-CfaE nanobodies provide broad cross-protection against major pathogenic enterotoxigenic Escherichia coli strains, with implications for vaccine design
Source: Sci Rep. 2021 Feb 2;11:2751. doi: 10.1038/s41598-021-81895-0 (PMC7854682; doi:10.1038/s41598-021-81895-0)
Supplement: Supplementary file 1 — Supplementary Information. [file 41598_2021_81895_MOESM1_ESM.pdf]

Anti-CfaE nanobodies provide broad cross-protection against major pathogenic Enterotoxigenic *Escherichia coli* strains, with implications for vaccine design

Alla Amcheslavsky[1], Aaron Wallace[1], Monir Ejemel[1], Qi Li[1], Conor T. McMahon[2], Matteo Stoppato[1], Serena Giuntini[1], Zachary A. Schiller[1], Jessica Pondish[1], Jacqueline R. Toomey[1], Ryan Schneider[1], Jordan Meisinger[1], Raimond Heukers[3], Andrew C. Kruse[2], Eileen M. Barry[4], Brian G. Pierce[5], Mark S. Klempner[1], Lisa A. Cavacini[1], Yang Wang*[1]

[1]MassBiologics, University of Massachusetts Medical School, Boston, Massachusetts, USA

[2]Department of Biological Chemistry and Molecular Pharmacology, Blavatnik Institute, Harvard Medical School, Boston, Massachusetts, USA

[3]QVQ B.V. Utrecht, the Netherlands

[4]Center for Vaccine Development, University of Maryland School of Medicine, Baltimore, Maryland, USA

[5]University of Maryland Institute for Bioscience and Biotechnology Research, Rockville, Maryland, USA

* Corresponding author

CORRESPONDING AUTHOR: Yang Wang

Yang.Wang@umassmed.edu

University of Massachusetts Medical School, MassBiologics, 460 Walk Hill Road, Boston, MA

Supplemental figure 1

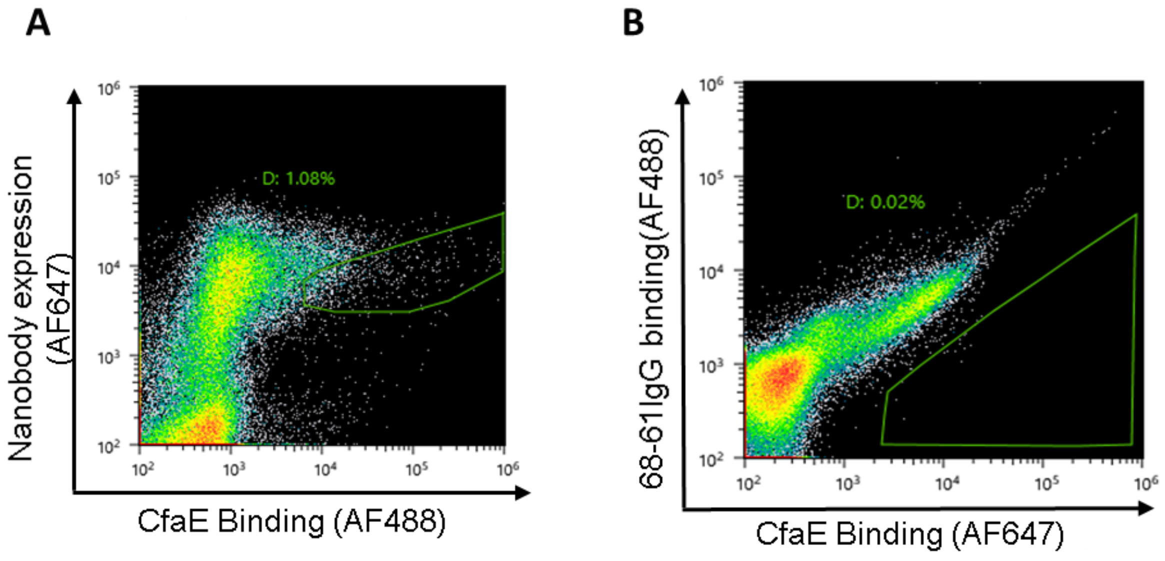

Supplemental figure 1. Strategy for selection of CfaE binding clones from yeast display library

(A) High-affinity binders were enriched by fluorescent activated cell sorting (FACS) with 300nM AlexaFluor488 labeled CfaE. Nanobody expression was monitored with AlexaFluor647 labeled anti-HA antibody.

(B) Competitive FACS screen was performed against a potent anti-CfaE human monoclonal antibody (HuMab), 68-61, to identify nanobodies specific to the receptor binding region of CfaE.

Supplemental figure 2

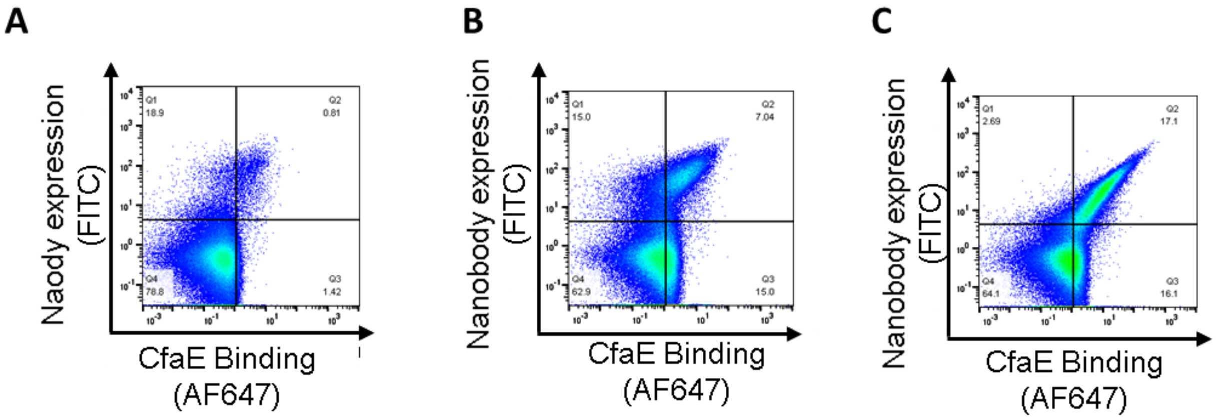

Supplemental figure 2. Yeast surface displayed clones show various binding affinities to CfaE protein

Flow analysis demonstrating examples of weak (A), moderate (B) and strong (C) binding of yeast displayed nanobodies to CfaE protein.

Supplemental table 1

	Rigid rods						Helical	Fibrillar	Nonfimbrial	Bundle-forming
	5a		5b			5c				
VHH	CS4	CS14	CS1	CS17	CS19	CS2	CS3	CS5	CS6	CS21
**2R215 IC_{100}(mM)**	5.5±1.37	13.3±0	13.3±0	1.31±1.02	9.44±3.96	8.75±2.84	5±1.68	1.04±0.06	3.89±1.82	1.25±0.59
**2R23 IC_{100}(mM)**	2.22±0.56	4.17±2.53	13.3±0	0.76±0.46	9.44±3.96	2.60±1.39	10±3.37	1.11±0.28	2.92±1.38	0.83±0.42
**1D7 IC_{100}(mM)**	7.29±3.49	6.65±0	5±1.68	0.31±0.10	5±1.7	0.94±0.63	3.6±1.68	0.97±0.74	3.61±1.72	0.97±0.37
**1H4 IC_{100}(mM)**	1.35±0.31	6.65±0	5.55±1.48	0.76±0.46	7.2±3.44	2.7±0.62	5±1.68	0.52±0.32	4.17±2.52	1.25±0.42

Supplemental table 1. Lead nanobodies are broadly cross-functional against multiple adhesins.

Four nanobodies were examined for activity in MRHA assay against seven strains representing class 5 of ETEC antigens and four other pathogenic fimbrial and non-fimbrial adhesins CS5, CS3, CS6 and CS21. Nanobodies showed broad protection against all tested strains with an IC_{100} ranging from 0.4125 to 13.3 µM.

Supplemental figure 3

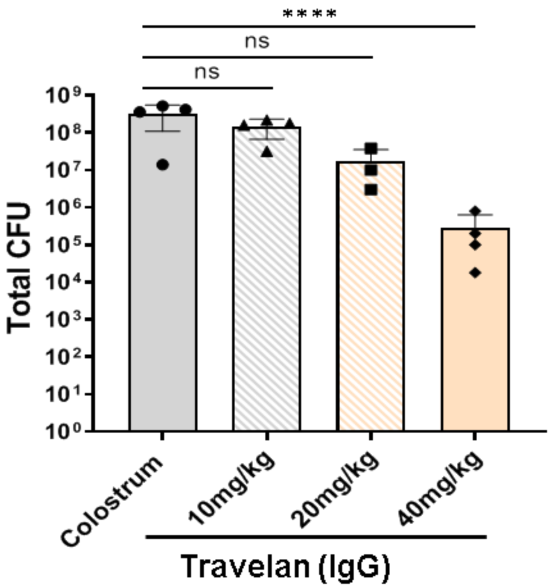

Supplemental figure 3. Effect of Travelan on ETEC colonization in one-hour pre-treatment model

Travelan was used at 10, 20 and 40mg/kg one hour before challenge with H10407. The dose of 40mg/kg resulted in 3.8 log reduction in colony numbers (*P*<0.0001).

Supplemental table 2

	L64	Y58	Y65	R67	Y156	R181	R182	Y183
2R215	++	++	+	++	++	++	++	++
1D7	-	-	+++	++	++	+	++	++

Supplemental Table 2. Residues required for VHH binding to conserved epitopes on class 5 colonization factors

Requirement of residues that were predicted by modeling as important for nanobody binding was examined in ELISA by substituting amino acids to alanine. ELISA results showed that mutating highly conserved residues Y58, L64, Y65, R67, R181, R182 and Y183 affected the binding of nanobodies to CfaE. +++ indicate very strong requirement (reduction in ELISA signal is more than 80% compared to wild type CfaE protein, ++ indicates strong requirement (50-80 % reduction in ELISA signal compared to wild type CfaE protein, + indicates weak requirement (20-50 % reduction in ELISA signal compared to wild type CfaE protein, - indicates no requirement (reduction in ELISA signal is less than 20% compared to wild type CfaE protein).

Figure 4B (original gel image)

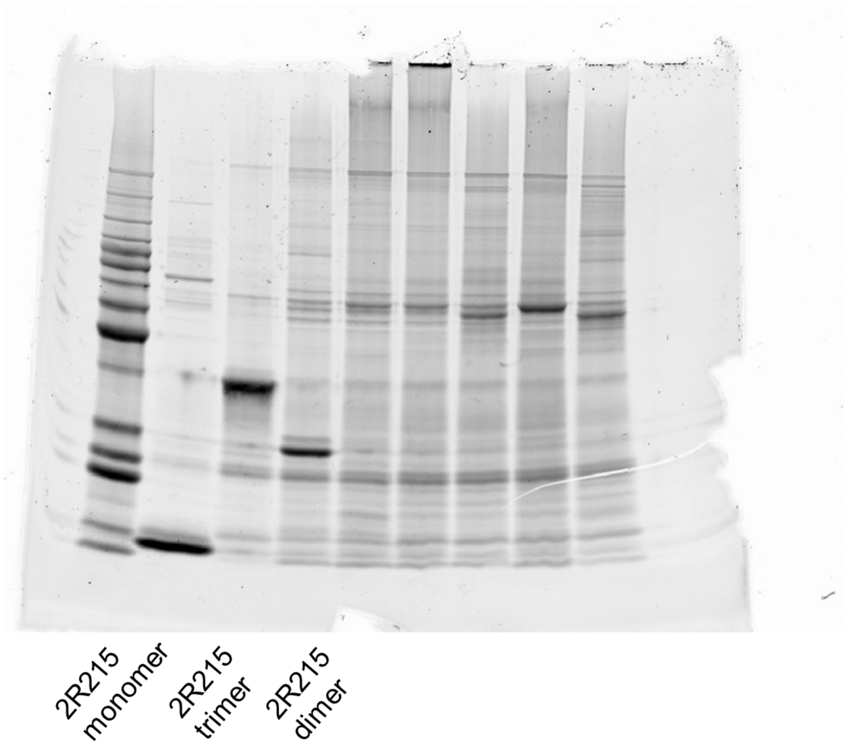

Figure 4B

Original, not cropped image gel, corresponding to Figure 4B in the manuscript is shown.

Figure 5B (original gel image)

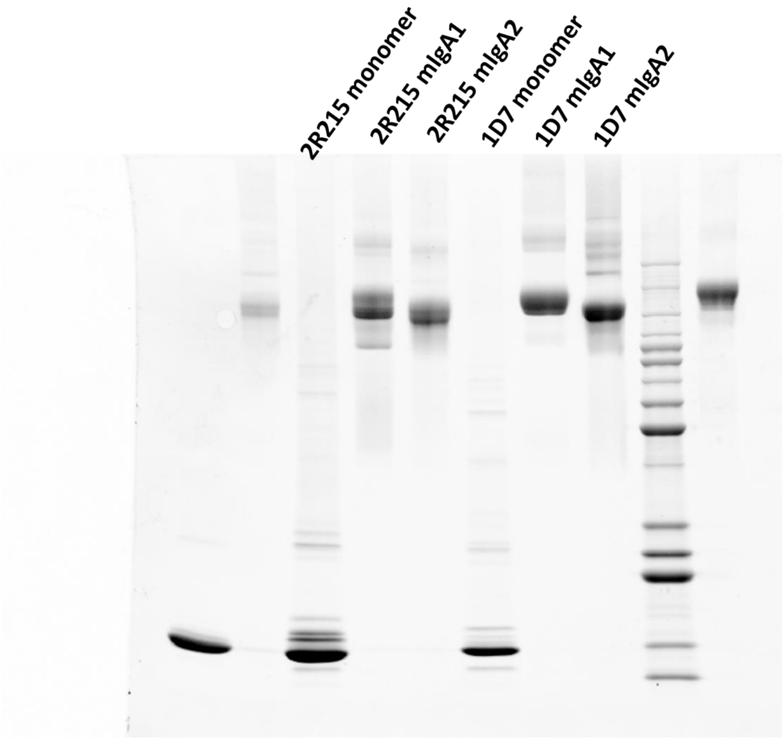

Figure 5B

Original, not cropped image gel, corresponding to Figure 5B in the manuscript is shown.
